# Registered nurses' experiences on job satisfaction in nursing home settings

**DOI:** 10.1002/nop2.2224

**Published:** 2024-06-23

**Authors:** Birgitta Jakobsson Larsson, Marie Mannberg, Ulrika Pöder, Mariann Hedström, Ann‐Christin Karlsson

**Affiliations:** ^1^ Department of Public Health and Caring Sciences Uppsala University Uppsala Sweden; ^2^ Department of Medical Sciences Uppsala University Uppsala Sweden

**Keywords:** job satisfaction, nursing home, qualitative research, registered nurse

## Abstract

**Aim:**

To describe what registered nurses' experience to be important to job satisfaction in nursing home settings.

**Design:**

This is a qualitative study based on data from individual interviews.

**Methods:**

Sixteen registered nurses working in nursing homes were interviewed, and their responses were analysed with systematic text condensation.

**Results:**

A total of six categories were developed to describe various aspects of job satisfaction among registered nurses at nursing homes: *meaningfulness is essential, to possess control and manageability is central, a possibility to balance daily challenges with professional development, supportive leadership is imperative, the nursing team's competence and companionship, and being confident in one's own profession*.

**Conclusion:**

In the present study, meaningfulness was essential to job satisfaction, and work was experienced to be meaningful and engaging when the demands were manageable, the workload controllable, and when the registered nurses felt supported by management and co‐workers. Conversely, if the demands were too high, the workload was beyond their control and the nurses felt unsupported, then the work felt meaningless and thus unsatisfactory.

## 1 INTRODUCTION

Working as a registered nurse (RN) in a nursing home is a complex and demanding job, requiring various skills (Cooper et al., 2017). The RN needs competence to recognise, reflect on and solve practical, medical and ethical problems that arise during the working day. The work tasks vary and include assessment, health promotion, risk management as well as end‐of‐life care (Kiljunen et al., 2017). Although the autonomous nature of the work as a RN in a nursing home has been described as important, it can also contribute to a feeling of loneliness due to the sparse amount of colleagues with whom one can discuss or seek advice (Carlson et al., 2014). Research has shown that the possibilities for discussions with colleagues is of great importance for RNs, regardless of the workplace (Ahlstedt, 2024).

## 2 BACKGROUND

The proportion of older people is expected to increase (United Nations, 2023). Simultaneously, there is a global nursing shortage (Marc et al., 2019). In Sweden, as in several other countries, the number of RNs working in long‐term care of older people has decreased in recent years (National Board of Health and Welfare, 2022). As the risk of developing various diseases increases with older age, so will the elderly population's complex need for advanced care continuity and long‐term planning. Stable and competent personnel is an important precondition for caring for this vulnerable group of frail older persons (McGilton et al., 2016). As a consequence of the nursing shortage, some of the RN tasks, such as medication administration, need to be delegated to the nursing staff. A recent Swedish report from the Health and Social Care Inspectorate (IVO), however, has highlighted alarming difficulties in recruiting nursing staff, revealing two major competence deficiencies i.e. lack of training and inadequate language skills (IVO, 2022). Taken together, recruiting problems constitute an actual risk for patients' safety in the long‐term care of older persons.

### 2.1 Organisation

Since 1992, the municipalities in Sweden have the responsibility for the long‐term care and services for the elderly, with the collective responsibility for healthcare services all the way up to the RN level, while the regions are responsible for medical interventions (Government Proposal 1990/91:14). The nursing staff who are responsible for the basic care are mainly subjected to the Social Services Act (SoL, 2001:453), while the RNs who are responsible for the specific nursing care are mainly subjected to the Health Care Act (HSL, 2017:30). Thus, these groups are principally governed by different laws. In Sweden, there are no educational requirements for the first‐line manager (hereinafter referred to as the manager) in elderly care (National Board of Health and Welfare, 2023), but managers at nursing homes usually have a degree in social service or nursing and are responsible for staff, finances and social services provided to the residents. Consequently, the manager may lack the training and knowledge to be able to support the nursing staff and RNs in nursing care decisions. This place demands on the RNs to supervise the nursing staff, and to ensure that they have the knowledge and skills to perform the nursing care that the residents need.

### 2.2 Job satisfaction

In two literature reviews, including RNs in long‐term care for older people, it was shown that both individual and organisational factors are of importance for job satisfaction (Aloisio et al., 2021; Lee, 2022), and that turnover among RNs is strongley associated with job dissatisfaction (Lee, 2022). Factors such as interprofessional collaboration, leadership, the relation with colleagues as well as with the older persons, favour the job satisfaction in RNs working in elderly care (Foa et al., 2020). Job satisfaction also affects the quality of care. A previous study, for example, found that there are fewer pressure ulcers and lower rates of hospital referrals in nursing homes where the RNs rate their job satisfaction as good (White et al., 2020).

Working in municipal elderly care requires both competence and experience to be able to meet the older persons' increased need for care. Previous studies have shown job satisfaction as immensely important in relation to nurse turnover rates, but also for the quality of care received by the residents. To our knowledge, however, no qualitative studies have been published on this subject in nursing home settings. Such knowledge is a prerequisite for creating conditions for an attractive, healthy workplace and reducing the RN turnover in nursing home settings, but above all, creating conditions so that the older persons receive the care they need. Therefore, we aim to describe what RNs' experience is important to job satisfaction in nursing home settings.

## 3 METHODS

### 3.1 Design

This is a qualitative study based on data from individual interviews. The consolidated criteria for reporting qualitative research (COREQ) were followed throughout the study (Tong et al., 2007).

### 3.2 Sampling and recruitment

This study was conducted in a county in central Sweden. The nursing homes in this area are staffed by assistant nurses or nurse's aides (hereinafter referred to as nursing staff) at all hours, and by RNs from 7 a.m. to 4 p.m. on weekdays. The RNs are responsible for nursing care and for informing/cooperating with the general practitioner (GP) about which residents need medical assessment. During evenings, nights and weekends, on‐call RNs are available for telephone consultations and emergency visits. Weekly on‐site consultations are made by a GP, employed by the county council. At other times, the RN has the possibility to consult with a GP by telephone.

A purposeful sampling was used to select participants (Malterud, 2012). First, three different municipalities were chosen, one large, where nursing homes are run by both public non‐profit and private for‐profit organisations and two small, where all nursing homes are run by the municipality. Thirty‐five department directors at nursing homes in three different municipalities were informed about the study and asked for help in recruiting RNs who were interested in participating in the study. A selection was sought, with a variation in age, sex, length of employment and both private and public employees. RNs from staffing agencies were excluded. Sixteen female RNs eligible from 11 different nursing homes contacted the second author (M.M) by phone or email, and consented to participate in an interview (Table 1). All participants were given an information letter about the study's purpose, informing that participation was voluntary, that data would be kept confidential and that withdrawal from the study was possible at any time, after which they gave their written informed consent. No participant withdrew.

**TABLE 1:** Demographics of participants (*n* = 16).

Employer	
Public non‐profit	8
Private for‐profit	8
Age	
25–35	6
36–45	4
46–55	4
56–65	2
Years as RN	
0–5	3
6–10	5
11–20	3
21–40	5
Years in current position	
0–1	7
2–3	7
4–5	2

### 3.3 Theoretical perspective

According to the job‐demand‐control‐support (JDCS) model, job satisfaction is achieved through a combination of demands, control and support. Negative stress occurs when the work demands are too high, and the employee's control and decision‐making influence is low. If the employee, instead, has the possibilities to make their own decisions to solve challenging assignments, a so‐called active work arises that contributes to control and strengthens the sense of manageability. Active work brings the opportunity for the employee to reveal confidence in their own ability, so‐called self‐efficacy, and a feeling of managing the demands, which is two important factors for job satisfaction. The third factor is support. A functioning support from colleagues, managers, family or friends has been shown to contribute to less stress (Karasek & Theorell, 1990). In this study, we have based some of the interview questions on the JDCS model and will discuss some findings accordingly. The model thus served as a framework for our interpretations of the results.

### 3.4 Data collection

Data were collected through individual face‐to‐face interviews, ranging from 23 to 60 min in length. Upon participants' request, most interviews took place in the RN's workplace. One interview was performed in a library in a reserved and secluded room. All interviews were conducted by the second author and took place in February and March 2017. A semi‐structured interview guide with open‐ended questions was used (Table 2), inspired by Karlsson et al. (2019) to highlight the RNs' overall experiences of job satisfaction. In addition, questions inspired by the JDCS model (Karasek & Theorell, 1990) were added to elucidate the RNs' job satisfaction based on demands, control and support. First, the RNs were asked questions concerning background characteristics, followed by questions of relevance to the aim of the study. Probing questions depended on the responses. All interviews were audio‐recorded and transcribed verbatim by the second author or by a secretary.

**TABLE 2:** Semi‐structured interview guide.

Main questions	Examples of probing questions
Your overall satisfaction with your work situation?^a^	Why do you feel that way?
Please tell me about a day when you are satisfied with your work^a^	How do you experience such a day? Describe your thoughts and emotions *during* the day Describe your thoughts and emotions *at the end* of the day
Please tell me about a day when you are dissatisfied with your work^a^	How do you experience such a day? Describe your thoughts and emotions *during* the day Describe your thoughts and emotions *at the end* of the day
What is important for you to feel satisfied with your work?^a^	
What is your perspective on the relation between job satisfaction and patient safety?^a^	Please develop
In relation to work satisfaction, can you give an example of^b^: The demands on your daily work performance? What control in your work means to you? What support in your work means to you?	Please describe the balance between demand, control and support at your workplace

*Note*: Questions inspired by ^a^Karlsson et al. (2019) and ^b^Karasek and Theorell (1990).

### 3.5 Data analysis

The transcripts were subjected to inductive data analysis through Systematic Text Condensation, a method for thematic analysis of qualitative data (Malterud, 2012). The analysis involved the method's four steps; (i) All transcriptions were read with a ‘birds‐eye‐perspective’ in order to elicit preliminary themes, and then (ii) text relevant to the purpose of the study was identified as meaning units and marked with a code, i.e. a label to connect related meaning units into code groups. During this step, the code groups and the preliminary themes were processed and adjusted in relation to each other, after which each code group represented a theme. In the third step (iii), the content of each code group was abstracted by condensation of the text, a process in which two of three aspects representing the content of each code group were identified. In accordance with the fourth and final analytic step (iv), each code group's content was further synthesised into categories, presented by a label and an analytic and descriptive text. Quotes were identified to illustrate the content, and participants' names were replaced with interview numbers. Reflexivity (Malterud, 2001) between all authors was considered throughout the data analysis to reduce any research bias. This was ensured by discussing possible preconceptions and genuine emanations from the data, especially as the second author has many years of experience as an RN specialised in elderly care.

### 3.6 Ethical considerations

Ethical approval was received from the Regional Ethical Review Board in Uppsala (Reg. no.2016/111, additional application approved 25 January 2017), and the study followed the Declaration of Helsinki.

### 3.7 Rigour

The trustworthiness of the study was ensured by considering credibility, transferability, and dependability (Polit et al., 2021). Credibility was supported by using individual interviews as the data collection method to obtain rich and detailed data. The interview guide ensured that the same questions were included in all interviews. Coding of the transcripts was independently performed by the second author (M.M), followed by peer debriefing with all the authors regarding the analysis in order to enhance the credibility of the results. The transferability of the study was enhanced with thick data obtained by interviewing RNs from either public non‐profit or private for‐profit nursing homes and by providing a detailed description of the participants and their experiences. For dependability, the exact procedure of data analysis was described according to Malterud (2012), and citations were used to support the results.

## 4 RESULTS

Six categories were developed to describe various aspects of RNs' job satisfaction at nursing homes: *meaningfulness is essential, to possess control and manageability is central, a possibility to balance daily challenges with professional development, supportive leadership is imperative, the nursing team's competence and companionship, and being confident in one's own profession*.

### 4.1 Meaningfulness is essential for job satisfaction

All the RNs expressed that they enjoyed working with people, and many had chosen the nursing profession to be able to make a difference in the lives of others. Working in nursing homes was perceived as meaningful, since it gave the opportunity to get to know both the residents and their relatives, an entirety that was appreciated by the RNs. The tasks were considered stimulating and varied, which enabled the RNs to use their knowledge; moreover, to perform well was deemed important for their well‐being. Gaining an appreciation for one's job made the work meaningful. The workplace was sometimes described as a second home and after a good day at work, many RNs felt they could leave work with a sense of satisfaction, knowing that they had done a good job.

I enjoy working with the elderly. I think it's great fun getting to know them and their relatives. The work tasks are fun too. And here at the nursing home, it is such a combination. Both administrative and some emergency situations, some wound dressings, a bit of everything. I guess that's why I enjoy it. (Interview 7)

Instead of leading nursing care, a lot of time was spent on administrative tasks in front of the computer. After a bad day, when nothing worked, the RNs felt frustrated, and irritated and expressed a desire to leave the nursing profession. A feeling of hopelessness and that healthcare was heading in the wrong direction led to a loss of both energy and enthusiasm. Work on such days felt less meaningful.

What do I really want to do? What can I do instead of what I am doing today? Can I work at this place? Can I work with this? Can I study a bit, so that I can work on something else? Hopelessness, frustration, fatigue. (Interview 5)

### 4.2 To possess control and manageability is central to job satisfaction

The importance of feeling capable, efficient and knowledgeable, as well as being involved in bedside nursing care, was emphasised. It was important to have control and be given the opportunity to work systematically on the planned tasks for the day. Keeping up and leaving nothing to chance were central to job satisfaction. This was achieved by meeting the residents every morning and by sitting down and talking to the nursing staff to ensure that work was done properly. If given the time needed for that, the work flowed, felt enjoyable and promoted patient safety.

You are satisfied if you feel that you have finished in time. You have done what you had planned for the day. It feels like you get to begin with a good starting position on the next day. You are satisfied; it feels calm and stable. (Interview 8)

High demands on documentation were perceived as disruptive, frustrating and time‐consuming, taking time away from performing nursing tasks as thoroughly as they desired. Moreover, knowing that tomorrow would look the same was expressed as burdensome and tiring. On messy days, the RNs felt worried about making mistakes. The stress could lead to lack of attention and missed tasks, thus posing a risk to patient safety. Feeling inadequate despite doing one's best contributed to anxiety and sleepless nights. Being alone with all the responsibility in these situations was one reason for wanting to leave the workplace.

I don't have time to be with everyone. I don't have time to do really well anywhere, but I just do what absolutely has to be done. It is truly horrible. (Interview 5)

The nursing shortage meant that the RNs had to cover for each other. Illness, education or holidays meant duplication of work without compensation. During the summer, the RNs often worked alone; moreover, with many substitutes in the nursing team, it often meant extra work.

It is frustrating that you always have to cover for each other. That there is no substitute. I'm one person, and I have to work for two people, twice as much, and you don't get double pay for that. (Interview 7)

### 4.3 A possibility to balance daily challenges with professional development contributes to job satisfaction

Working in a nursing home involved many responsibilities, which presented challenges, ranging from administrative tasks; and motivating, supervising and teaching residents, staff and students; to emergency interventions. Education and personal development were emphasised as important and as a good foundation for making RNs feel confident and more able to handle stressful situations. Keeping up and learning new things was stimulating and important in order to meet medical challenges. Therefore, the RNs felt it was important that the management prioritised, offered time, and supported improvement work by using the RNs' expertise. Quality improvement work contributed to job satisfaction and an improved psychosocial work environment; however, it was difficult, due to time constraints and high staff turnover. Tasks piled up, thus resignation and the feeling of not being able to influence one's situation reduced job satisfaction. Instead of developing the work, time was spent trying to catch up with work that was not done. Without the opportunity to advance based on their nursing skills and with what they were interested in, their work felt boring and contributed to RNs wanting to change their workplaces.

You always need new experiences; you need to develop. If you don't develop at the workplace, that you have time for it and the manager is responsive to it, then you end up having to do it yourself, and then you might change workplaces. (Interview 3)

One description of working at nursing homes was that the days were about problem‐solving, that the work was like climbing a mountain to get a better view, and when you could solve the situation, it felt good. However, the RNs stated that the situation for the nursing staff was not always optimal and realised that it was a challenge for them to perform a good job, given the conditions. Educating and supervising in a more structured way was a stated wish to improve job satisfaction for all.

### 4.4 Supportive leadership is imperative for job satisfaction

Leadership and good cooperation with the manager were considered as the most important aspects for job satisfaction. It was important that the manager saw, confirmed and gave positive feedback on the work tasks performed by the RNs. If the work task was too burdensome, it was of great importance that the manager paid attention to it. However, it was not enough to just listen; the RNs desired active actions from the manager to reduce their workload. This could mean a consensus on certain issues or working towards a common goal. A clear leader who was nevertheless accepting of others' knowledge and experiences made the RNs feel more responsible towards their work.

It's important that you work together with the manager and not against each other. (Interview 6)

Many RNs emphasised that it was easier to consult with a manager who was also an RN because he or she had an understanding of and could facilitate the RNs' work situation. For example, there was a perception that managers without nursing education had a lack of understanding of what a delegation to the nursing staff meant for the responsible RN on duty.

… managers who do not have nursing training have less understanding of our tasks than those who have worked as nurses before. They have an easier time understanding when we say that we don't have time for many tasks than those who only have some social work training. (Interview 11)

The RNs found it difficult to carry out their work tasks when the managers handed over supervisory responsibilities. When problems arose with the nursing staff, or when difficult conversations with the nursing staff were left to the RNs, although it was not described as their responsibility, they often felt alone. They wanted the manager to stand up for them and show commitment when, for example, routines were not followed, which, in turn, raised concerns that it could negatively affect patient safety. They felt powerless when the manager did not employ trained staff, or when the nursing staff did not follow directions or refused to perform tasks such as wound dressings. The RNs felt that they sometimes needed to act as police officers, monitoring that the nursing staff carried out their duties – tasks that were considered to be in the hands of the manager.

I would like to have better support, communication and collaboration with my manager. Where she is involved in what happens in my department and what my workload is like. (Interview 2)

### 4.5 The nursing team's competence and companionship contribute to job satisfaction

The nurse colleagues, in particular, were important to the RNs. Flexibility, complementing and helping each other with difficult medical judgements and ethical dilemmas were essential to thrive in the nursing team. It was common to contact each other in their spare time after a stressful day if support was needed. Therefore, the RNs felt sad when colleagues quit, as social contact was lost. The RNs also emphasised loyalty between colleagues, i.e. not to hand over tasks to the on‐call RN in the evening, but to make sure to finish the work before going home and thus facilitating those who took over.

We are very good in the working group. I think that we are good at praising each other; how well something turned out and so on. If there is something bothering you, we sort it out. (Interview 10)

The RNs valued having a good relationship with everyone in the team and appreciated when the nursing staff created a dialogue and made suggestions for solutions. It was important to have competent staff because tasks could then be delegated, which was a support for the RNs. They appreciated and wanted to have time for thought and reflection after a shift and to have a chat with the nursing staff. It was a way to learn from the different situations that arose during the shift and was viewed as a time for recovery.

So this helping each other, talking to each other all the time. De‐brief if there is anything there; talk about the incident, so you don't take them home with you. (Interview 11)

It was considered important for job satisfaction that everyone in the team knew their tasks and how things worked. When the nursing team was short‐staffed, with no staff to delegate to, or when there were many substitutes or if there were other problems in the team that made it feel unstable, nursing care was negatively affected, resulting in frustration for the RNs. A major problem that was highlighted was when the nursing staff did not carry out the RN's directions regarding temperature checks, blood sugar checks, or post‐treatment evaluations. They saw themselves as being grumpy, nagging and whining when this needed to be pointed out. The RNs were sometimes asked to help out with bedside care to relieve the nursing staff, but then their own tasks suffered and were not carried out according to the plan. The disappointment of not getting support from the nursing staff led to an increased workload for the RNs and affected job satisfaction negatively.

If I have to check constantly on things that I have ordered, whether it is done or not done, it becomes stressful. When someone hasn't done what's written, or someone says they didn't know when it's very clear, then I get very frustrated. (Interview 11)

### 4.6 Being confident in one's own profession contributes to job satisfaction

RNs who had worked for a long time, both as a RN and at the same nursing home, felt confident that they had developed knowledge and experience that allowed them to organise their work as they wished. Being able to plan one's own time led to a sense of freedom, and it felt reassuring to know how to handle unexpected situations. Experienced RNs were keen to use their knowledge and help find solutions to problems together with the nursing team. It also gave job satisfaction to relieve colleagues who needed support.

This clinical look that you talk about – that you actually learn it. It sounds vague from the start, that you can just look at a patient and tell how they are feeling and what is wrong, but you learn it. And there is some satisfaction in seeing that you are starting to know your stuff. (Interview 8)

Having easy‐to‐follow routines to lean on provided a sense of safety, which felt especially important as staff turnover was high. It was considered important for job satisfaction that everyone in the team knew their tasks and how things worked. Being anchored in the routines and knowing that everyone worked actively based on the established routines facilitated the work. Therefore, it was perceived as troubling when written routines were difficult to find. It was time‐consuming to look for these or not to get an answer from the manager about what was applicable. Unclear routines meant there was room for personal values and opinions, which resulted in the RNs and the nursing staff working in different ways.

If everyone knows their duties and it works, I think it brings more joy and satisfaction. Then you feel better. (Interview 11)

Being available to the nursing staff, the doctor and the manager means that one is everywhere, and helps to solve problems that come up. However, the RNs themselves were often overloaded with work and did not have the time or the opportunity to support their colleagues. The feeling could be described as a roller coaster, where the work was sometimes great fun and where the RNs felt proud of their profession, but at the same time, they felt that they would probably not be able to cope in the long run due to the heavy workload, with the risk of ill health.

The sole responsibility. Plus, now when you hand over and you report when you go home, you can't be sure that things are done. And if something more advanced is needed, the people who come then are from the emergency department; they don't know the residents. (Interview 9)

## 5 DISCUSSION

In this qualitative study on what RNs experienced to be important in relation to their job satisfaction when working in nursing homes, we identified six categories: meaningfulness is essential, possessing control and manageability is central, a possibility to balance daily challenges with professional development, supportive leadership is imperative, the nursing team's competence and companionship, and being confident in one's own profession. During the interviews, the RNs were asked to give examples of job satisfaction in relation to demands, control and support, inspired by Karasek's job‐demand‐control‐support (JDCS) model (Karasek & Theorell, 1990), and some results will be discussed in relation to these concepts.

A sense of meaningfulness when making a difference in others' lives was perceived as essential in relation to job satisfaction. Most RNs mentioned that they had chosen the nursing profession for this cause and enjoyed using their skills and providing good care, as well as the opportunities to form relations with the residents and their relatives. The special relations developed between the resident and the staff have been described as important for job satisfaction in previous studies (Carlson et al., 2014; Marshall et al., 2020; Min et al., 2022). A good and meaningful relationship with residents and their families helped the RN to gain strength (Foa et al., 2020; Min et al., 2022) and reduced work‐related stress (Foa et al., 2020).

Even though our results show that RNs appreciate the variety in their job, it was also important to have control. To possess control and manageability during the workday was described as central to job satisfaction. The need for control over their work is not specific to RNs in nursing homes, the importance of control has also been highlighted by RNs working at hospitals (Karlsson et al., 2019). Control for the RNs meant having the knowledge and possibility to perform nursing tasks. The RNs were frustrated when the work could not be done as thoroughly as desired due to a lack of time or an overload of administrative tasks. When RNs experienced being autonomous and competent for their tasks, the work felt controllable, which according to Karasek and Theorell (1990), is a prerequisite for job satisfaction and promotes patient safety, which is in accordance with the results of this present study as well as previous ones (Inoue et al., 2017; Karlsson et al., 2019). On the other hand, not having control caused anxiety and fear of making mistakes. To keep up‐to‐date and to learn new things contributed to a foundation, that made it possible to balance daily challenges with professional development. This need for lifelong learning, has also been emphasised for nursing in municipal home health care (Claesson et al., 2020).

Supportive leadership was imperative for the RNs' job satisfaction, which is congruent with earlier studies on RN's job satisfaction regardless of workplace (Ahlstedt, 2024; Karlsson et al., 2019; Schwendimann et al., 2016). Experiencing well‐functioning support from the manager and colleagues, and a sense of community in the work group contribute to reducing stress, and affect the experience of job satisfaction positively (Karasek & Theorell, 1990). In the present study, the RNs also highlighted the need for a manager who was also an RN, as they were considered to be able to better provide support and advice in difficult situations and more easily familiarise themselves with the RNs' work situation.

The nursing team's competence and companionship were important, and it was appreciated when the nursing staff came up with proposed solutions and dialogue was created. The nurse colleagues in particular were important to the RNs job satisfaction. To be able to ask questions, discuss and learn from each other is important (Ahlstedt, 2024; Schwendimann et al., 2016), regardless of whether working in nursing homes or hospitals. A support in the RNs' work would be the ability to delegate tasks to other nursing staff, but it required stability and competence in the nursing team to make this possible. A report from The Health and Social Care Inspection (IVO), shows that there are major deficiencies in the competence, training and language skills of the nursing staff (IVO, 2022). Delegation also makes it difficult for the RNs to have full control over the care that the residents receive. When the nursing team was unstable, and the delegated tasks were not performed, the RNs felt frustrated and powerless.

RNs who had developed knowledge and experience were confident in their own profession and felt assured that they could handle unexpected situations and support colleagues, which contributed to job satisfaction. However, despite several years of professional experience, the RNs could also experience some anxiety about the lonely work in a nursing home. The work as a RN in nursing homes is indeed a complex and demanding job, where the RN need to possess many different skills (Cooper et al., 2017; Kiljunen et al., 2017). RNs in our study perceived that their work in nursing home entailed a lot of demands, such as demands for good and safe care, as well as demands from residents, relatives and management. The manager's demands, particularly related to covering vacancies and working with unskilled nursing staff, were occasionally perceived as excessively high. In situations where the opportunity to make independent decisions was limited, stress and job satisfaction were adversely affected, which is in accordance with other studies (Ahlstedt, 2024; Karlsson et al., 2019).

The study highlights, in accordance with the JDCS model (Karasek & Theorell, 1990), that demands, control as well as support is of importance for job satisfaction among RNs in nursing home settings. Although meaningfulness is not a concept in the JDCS‐model, it was decisive for the RNs experiences of job satisfaction, which is important to take in account in future studies to gain a deeper insight into what RNs experience to be of importance for their job satisfaction.

### 5.1 Limitations

The findings of our study have certain limitations. Although we sought to have variations by including RNs from both public non‐profit and private for‐profit nursing homes, RNs of different ages and years as an RN and with a difference in years in current position, only female RNs participated voluntarily, which could limit the transferability of the findings. Further studies that enrol and study male RNs' experiences of job satisfaction in nursing homes are needed. During the data analysis, we strived for an inductive approach, although it is likely that the concepts of the JDCS model to some extent influenced the data analysis, especially since some of the interview questions were based on the model. Even so, we found the model appropriate when developing relevant interview questions, and believe our study benefitted from this approach. The data were collected 6 years ago; consequently, they may not reflect the views of today's RNs in elderly care in relation to the purpose of this study. However, the study still has a news value, as the findings are in line with recent studies from other contexts. To our knowledge, there are no similar qualitative studies reflecting RNs' job satisfaction in nursing homes in relation to control, demand and support in order to experience one's work as manageable and meaningful.

### 5.2 Implications for the profession and/or patient care

The results can be used as a basis for developing a healthy work environment for registered nurses working in nursing homes. An improved job satisfaction for registered nurses can positively affect the quality of care at nursing homes. The current findings also provide valuable insights, which can be used by managers for discussions with registered nurses working in nursing homes to enhance their meaningfulness, well‐being and job satisfaction based on demand, control and support.

## 6 CONCLUSION

This study highlights several aspects of the importance of job satisfaction among RNs in nursing home settings. The relationship with the residents and their relatives was valued. In addition, being able to use one's knowledge to do something good and to be appreciated for one's work contributed to a sense of job satisfaction and helped the RNs to gain strength. When the demands were manageable, the workload controllable and the RNs felt supported by management and co‐workers, then the work felt meaningful and engaging. Conversely, when demands were too high, the workload uncontrollable and the RNs felt unsupported, then the work felt meaningless and thus unsatisfactory. Findings from this study may have potential implications for management policy to support RNs in nursing home settings, based on demand, control and support and take into account its effect on RNs' job satisfaction and well‐being.

## AUTHOR CONTRIBUTIONS

Marie Mannberg, Ulrika Pöder and Mariann Hedström determined the design and aim of the study. Marie Mannberg led the interview process and the data analysis. Birgitta Jakobsson Larsson had the main responsibility for writing the manuscript, together with Ann‐Christin Karlsson. All authors were involved in the interpretation of the data. All authors reviewed and edited the manuscript and approved the final version of the manuscript.

## FUNDING INFORMATION

This research received no specific grant from any funding agency in the public, commercial or not‐for‐profit sectors.

## CONFLICT OF INTEREST STATEMENT

The authors declare no conflicts of interest.

## RESEARCH ETHICS COMMITTEE APPROVAL

Ethical approval was received from the Regional Ethical Review Board in Uppsala (Reg. no. 2016/111, additional application approved 25 January 2017).

## PATIENT OR PUBLIC CONTRIBUTION

No patients or members of the public were involved in the data collection (interviews).

## Data Availability

Not available due to ethical restrictions.

## References

[nop22224-bib-0001] Ahlstedt, C. (2024). *Registered nurses' work motivation and intention to stay at the workplace* (Publication Number 2020) [Doctoral thesis, comprehensive summary, Acta Universitatis Upsaliensis]. DiVA. http://urn.kb.se/resolve?urn=urn:nbn:se:uu:diva‐523652

[nop22224-bib-0002] Aloisio, L. D. , Coughlin, M. , & Squires, J. E. (2021). Individual and organizational factors of nurses' job satisfaction in long‐term care: A systematic review. International Journal of Nursing Studies, 123, 104073. 10.1016/j.ijnurstu.2021.104073 34536909

[nop22224-bib-0003] Carlson, E. , Ramgard, M. , Bolmsjo, I. , & Bengtsson, M. (2014). Registered nurses' perceptions of their professional work in nursing homes and home‐based care: A focus group study. International Journal of Nursing Studies, 51(5), 761–767. 10.1016/j.ijnurstu.2013.10.002 24176717

[nop22224-bib-0004] Claesson, M. , Jonasson, L. L. , Lindberg, E. , & Josefsson, K. (2020). What implies registered nurses' leadership close to older adults in municipal home health care? A systematic review. BMC Nursing, 19, 30. 10.1186/s12912-020-00413-1 32336946 PMC7171838

[nop22224-bib-0005] Cooper, E. , Spilsbury, K. , McCaughan, D. , Thompson, C. , Butterworth, T. , & Hanratty, B. (2017). Priorities for the professional development of registered nurses in nursing homes: A Delphi study. Age and Ageing, 46(1), 39–45. 10.1093/ageing/afw160 28181630

[nop22224-bib-0006] Foa, C. , Guarnieri, M. C. , Bastoni, G. , Benini, B. , Giunti, O. M. , Mazzotti, M. , Rossi, C. , Savoia, A. , Sarli, L. , & Artioli, G. (2020). Job satisfaction, work engagement and stress/burnout of elderly care staff: A qualitative research. Acta Biomed, 91(12‐S), e2020014. 10.23750/abm.v91i12-S.10918 PMC8023104 33263342

[nop22224-bib-0007] Inoue, T. , Karima, R. , & Harada, K. (2017). Bilateral effects of hospital patient‐safety procedures on nurses' job satisfaction. International Nursing Review, 64(3), 437–445. 10.1111/inr.12336 28133718

[nop22224-bib-0008] IVO (The Health and Social Care Inspectorat) . (2022). Supervision of medical care and treatment at special residences for the elderly. Partial report of results at national level regarding the municipalities' health care [Tillsyn av medicinsk vård och behandling vid särskilda boenden för äldre. Delredovisning av resultat på nationell nivå avseende kommunernas hälso‐ och sjukvård] . https://www.ivo.se/aktuellt/publikationer/rapporter/tillsyn‐av‐medicinsk‐vard‐och‐behandling‐vid‐sarskilda‐boende‐for‐aldre‐sabo/ (In Swedish).

[nop22224-bib-0009] Karasek, R. , & Theorell, T. (1990). Healthy work: Stress, productivity, and the reconstruction of working life. Basic Books.

[nop22224-bib-0010] Karlsson, A. C. , Gunningberg, L. , Backstrom, J. , & Poder, U. (2019). Registered nurses' perspectives of work satisfaction, patient safety and intention to stay ‐ A double‐edged sword. Journal of Nursing Management, 27(7), 1359–1365. 10.1111/jonm.12816 31211908

[nop22224-bib-0011] Kiljunen, O. , Valimaki, T. , Kankkunen, P. , & Partanen, P. (2017). Competence for older people nursing in care and nursing homes: An integrative review. International Journal of Older People Nursing, 12(3), e12146. 10.1111/opn.12146 28032436

[nop22224-bib-0012] Lee, J. (2022). Nursing home nurses' turnover intention: A systematic review. Nursing Open, 9(1), 22–29. 10.1002/nop2.1051 34811952 PMC8685779

[nop22224-bib-0013] Malterud, K. (2001). Qualitative research: Standards, challenges, and guidelines. Lancet, 358(9280), 483–488. 10.1016/s0140-6736(01)05627-6 11513933

[nop22224-bib-0014] Malterud, K. (2012). Systematic text condensation: A strategy for qualitative analysis. Scandinavian Journal of Public Health, 40(8), 795–805. 10.1177/1403494812465030 23221918

[nop22224-bib-0015] Marc, M. , Bartosiewicz, A. , Burzynska, J. , Chmiel, Z. , & Januszewicz, P. (2019). A nursing shortage ‐ A prospect of global and local policies. International Nursing Review, 66(1), 9–16. 10.1111/inr.12473 30039849

[nop22224-bib-0016] Marshall, E. G. , Power, M. , Edgecombe, N. , & Andrew, M. K. (2020). Above and beyond: A qualitative study of the work of nurses and care assistants in long term care. Work, 65(3), 509–516. 10.3233/WOR-203105 32116270

[nop22224-bib-0017] McGilton, K. S. , Bowers, B. J. , Heath, H. , Shannon, K. , Dellefield, M. E. , Prentice, D. , Siegel, E. O. , Meyer, J. , Chu, C. H. , Ploeg, J. , Boscart, V. M. , Corazzini, K. N. , Anderson, R. A. , & Mueller, C. A. (2016). Recommendations from the international consortium on professional nursing practice in long‐term care homes. Journal of the American Medical Directors Association, 17(2), 99–103. 10.1016/j.jamda.2015.11.001 26712302

[nop22224-bib-0018] Min, D. , Cho, E. , Kim, G. S. , Lee, K. H. , Yoon, J. Y. , Kim, H. J. , & Choi, M. H. (2022). Factors associated with retention intention of registered nurses in Korean nursing homes. International Nursing Review, 69(4), 459–469. 10.1111/inr.12754 35413132 PMC9790496

[nop22224-bib-0019] National Board of Health and Welfare . (2022). Appendix 1. Results of supply and demand for the 22 the identification professions. National planning support 2022 [Bilaga 1. Resultat av tillgång och efterfrågan på de 22 legitimationsyrkena. Nationella planeringsstödet 2022] . https://www.socialstyrelsen.se/globalassets/sharepoint/dokument/artikelkatalog/ovrigt/2022‐2‐7759.pdf (In Swedish).

[nop22224-bib-0020] National Board of Health and Welfare . (2023). Introduce a limit on the number of employees and education requirements for first‐line managers in elderly care. Assessments and analysis of benefits and disadvantages [Införa en gräns för antal anställda och utbildningskrav för första linjens chefer i äldreomsorgen. Bedömningar och analyser av fördelar och nackdelar] . https://www.socialstyrelsen.se/globalassets/sharepoint‐dokument/artikelkatalog/ovrigt/2023‐3‐8413.pdf (In Swedish).

[nop22224-bib-0021] Polit, D. F. , & Beck, C. T. (2021). Essentials of nursing research: Appraising evidence for nursing practice . Wolters Kluwer Health.

[nop22224-bib-0022] Sveriges Riksdag . Social Services Act (SoL, 2001:453) [Socialtjänstlagen] . https://www.riksdagen.se/sv/dokument‐och‐lagar/dokument/svensk‐forfattningssamling/socialtjanstlag‐2001453_sfs‐2001‐453/ (In Swedish).

[nop22224-bib-0023] Schwendimann, R. , Dhaini, S. , Ausserhofer, D. , Engberg, S. , & Zuniga, F. (2016). Factors associated with high job satisfaction among care workers in Swiss nursing homes ‐ A cross sectional survey study. BMC Nursing, 15, 37. 10.1186/s12912-016-0160-8 27274334 PMC4895903

[nop22224-bib-0024] Sveriges Riksdag . Government proposal 1990/91:14 about the responsibility for service and care for the elderly and disabled, etc [Regerings proposition 1990/91:14 om ansvaret för service och vård till äldre och handikappade m.m] . https://www.riksdagen.se/sv/dokument‐och‐lagar/dokument/proposition/om‐ansvaret‐for‐service‐och‐vard‐till‐aldre‐och_ge0314/html/ (In Swedish).

[nop22224-bib-0025] Sveriges Riksdag . Health and Medical Care Act (HSL, 2017:30) . Ministry of Health and Social Affairs [Socialdepartementet]. https://www.riksdagen.se/sv/dokument‐ochlagar/dokument/svensk‐forfattningssamling/halso‐och‐sjukvardslag‐201730_sfs‐2017‐30/ (In Swedish).

[nop22224-bib-0026] Tong, A. , Sainsbury, P. , & Craig, J. (2007). Consolidated criteria for reporting qualitative research (COREQ): A 32‐item checklist for interviews and focus groups. International Journal for Quality in Health Care, 19(6), 349–357. 10.1093/intqhc/mzm042 17872937

[nop22224-bib-0027] United Nations . (2023). World Social Report 2023; Leaving no one behind in an ageing world . https://www.un.org/development/desa/pd/sites/www.un.org.development.desa.pf/undesa_pd_2023_wsr‐fullreport.pdf

[nop22224-bib-0028] White, E. M. , Aiken, L. H. , Sloane, D. M. , & McHugh, M. D. (2020). Nursing home work environment, care quality, registered nurse burnout and job dissatisfaction. Geriatric Nursing, 41(2), 158–164. 10.1016/j.gerinurse.2019.08.007 31488333 PMC7051884

